# Supplementary material for: From sequence to enzyme mechanism using multi-label machine learning
Source: BMC Bioinformatics. 2014 May 19;15:150. doi: 10.1186/1471-2105-15-150 (PMC4229970; doi:10.1186/1471-2105-15-150)
Supplement: Additional file 2 — Java code of ml2db. Additional file ml2db_code.tar.gz contains the Java source code to run the multi-label machine learning experiments and save the results to database. The code’s Javadoc is included. [file 1471-2105-15-150-S2.zip › additional file 2/ml2db/ml2db/doc/mulan/data/package-tree.html]

mulan.data Class Hierarchy


JavaScript is disabled on your browser.


- Overview
- Package
- Class
- Use
- Tree
- Deprecated
- Index
- Help

- Prev
- Next

- Frames
- No Frames

- All Classes

# Hierarchy For Package mulan.data

Package Hierarchies:

- All Packages

## Class Hierarchy

- java.lang.Object
  - mulan.data.ConditionalDependenceIdentifier (implements mulan.data.LabelPairsDependenceIdentifier, java.io.Serializable)
  - mulan.data.ConverterCLUS
  - mulan.data.ConverterLibSVM
  - mulan.data.DataUtils
  - mulan.data.GreedyLabelClustering (implements mulan.data.LabelClustering, java.io.Serializable)
  - mulan.data.IterativeStratification (implements mulan.data.Stratification, weka.core.TechnicalInformationHandler)
  - mulan.data.LabelNodeImpl (implements mulan.data.LabelNode, java.io.Serializable)
  - mulan.data.LabelPowersetStratification (implements mulan.data.Stratification, weka.core.TechnicalInformationHandler)
  - mulan.data.LabelsBuilder
  - mulan.data.LabelSet (implements java.lang.Comparable<T>, java.io.Serializable)
  - mulan.data.LabelsMetaDataImpl (implements java.io.Externalizable, mulan.data.LabelsMetaData, java.io.Serializable)
  - mulan.data.LabelsPair (implements java.lang.Comparable<T>, java.io.Serializable)
  - mulan.data.MultiLabelInstances (implements java.io.Serializable)
  - mulan.data.Statistics (implements java.io.Serializable, weka.core.TechnicalInformationHandler)
  - java.lang.Throwable (implements java.io.Serializable)
    - java.lang.Exception
      - mulan.core.MulanException
        - mulan.data.InvalidDataFormatException
        - mulan.data.LabelsBuilderException
      - java.lang.RuntimeException
        - mulan.core.MulanRuntimeException
          - mulan.data.DataLoadException
  - mulan.data.UnconditionalChiSquareIdentifier (implements mulan.data.LabelPairsDependenceIdentifier, java.io.Serializable)

## Interface Hierarchy

- mulan.data.LabelClustering
- mulan.data.LabelNode
- mulan.data.LabelPairsDependenceIdentifier
- mulan.data.LabelsMetaData
- mulan.data.Stratification

- Overview
- Package
- Class
- Use
- Tree
- Deprecated
- Index
- Help

- Prev
- Next

- Frames
- No Frames

- All Classes
